# Respiration of Microbiota-Derived 1,2-propanediol Drives *Salmonella* Expansion during Colitis

**DOI:** 10.1371/journal.ppat.1006129

**Published:** 2017-01-05

**Authors:** Franziska Faber, Parameth Thiennimitr, Luisella Spiga, Mariana X. Byndloss, Yael Litvak, Sara Lawhon, Helene L. Andrews-Polymenis, Sebastian E. Winter, Andreas J. Bäumler

**Affiliations:** 1 Department of Medial Microbiology and Immunology, School of Medicine, University of California Davis, Davis, CA, United States of America; 2 Department of Microbiology, Faculty of Medicine, Chiang Mai University, Chiang Mai, Thailand; 3 Department of Microbiology, University of Texas Southwestern Medical Center, Dallas, TX, United States of America; 4 Department of Veterinary Pathobiology, College of Veterinary Medicine, Texas A&M University, College Station, TX, United States of America; 5 Department of Microbial Pathogenesis and Immunology, College of Medicine, Texas A&M University Health Science Center, Bryan, TX, United States of America; University of Texas Southwestern Medical Center, UNITED STATES

## Abstract

Intestinal inflammation caused by *Salmonella enterica* serovar Typhimurium increases the availability of electron acceptors that fuel a respiratory growth of the pathogen in the intestinal lumen. Here we show that one of the carbon sources driving this respiratory expansion in the mouse model is 1,2-propanediol, a microbial fermentation product. 1,2-propanediol utilization required intestinal inflammation induced by virulence factors of the pathogen. *S*. Typhimurium used both aerobic and anaerobic respiration to consume 1,2-propanediol and expand in the murine large intestine. 1,2-propanediol-utilization did not confer a benefit in germ-free mice, but the *pdu* genes conferred a fitness advantage upon *S*. Typhimurium in mice mono-associated with *Bacteroides fragilis* or *Bacteroides thetaiotaomicron*. Collectively, our data suggest that intestinal inflammation enables *S*. Typhimurium to sidestep nutritional competition by respiring a microbiota-derived fermentation product.

## Author Summary

*Salmonella enterica* serovar Typhimurium induces intestinal inflammation to induce the generation of host-derived respiratory electron acceptors, thereby driving a respiratory pathogen expansion, which aids infectious transmission by the fecal oral route. However, the identity of nutrients serving as electron donors to enable *S*. Typhimurium to edge out competing microbes in the competitive environment of the gut are just beginning to be worked out. Here we demonstrate that aerobic and anaerobic respiratory pathways cooperate to promote growth of Salmonella on the microbial fermentation product 1,2-propanediol. We propose that pathogen-induced intestinal inflammation enables *Salmonella* to sidestep nutritional competition with the largely anaerobic microbiota by respiring a microbe-derived metabolite that cannot be consumed by fermentation.

## Introduction

*Salmonella enterica* serovar Typhimurium (*S*. Typhimurium) is a common cause of food poisoning. Upon ingestion, the pathogen enters the intestinal epithelium using the invasion-associated type III secretion system (T3SS-1) [1] and deploys a second type III secretion system (T3SS-2) to survive in host tissue [2]. This virulence strategy results in acute intestinal inflammation and diarrhea [3]. Interestingly, gut inflammation increases the abundance of the pathogen within the gut-associated microbial community [4] by generating a respiratory nutrient-niche (reviewed in [5]). One respiratory electron acceptor generated as a byproduct of the inflammatory response is tetrathionate, which confers a luminal growth advantage upon *S*. Typhimurium in a mouse colitis model [6] by enabling the pathogen to consume ethanolamine in the gut [7].

Genes involved in tetrathionate respiration and ethanolamine-utilization are intact in *Salmonella* serovars associated with gastroenteritis in humans, but are often disrupted in *Salmonella* serovars associated with extraintestinal disease [8–10]. Another pathway often disrupted in *Salmonella* serovars associated with extraintestinal disease is the utilization 1,2-propanediol [8–10], which is produced during the fermentation of rhamnose or fucose [11]. Genes required for the degradation of 1,2-propanediol are encoded by the *porR pduF pduABCDEGHJKLMNOPQSTUVWX* gene cluster [12], a DNA region conserved among *S*. *enterica* serovars, but absent from the closely related species *S*. *bongori* [13–15]. Both ethanolamine and 1,2-propanediol-utilization proceeds through a pathway that requires a microcompartment, a respiratory electron acceptor and the cofactor cobalamin [16]. Cobalamin biosynthesis genes are only expressed when *S*. Typhimurium is cultured under anaerobic or microaerobic conditions [17, 18]. Under anaerobic conditions, tetrathionate can serve as an electron acceptor to support *in vitro* growth of *S*. Typhimurium on 1,2-propanediol and ethanolamine by using endogenously synthesized cobalamin [19]. Based on these observations it has been proposed that tetrathionate respiration might enable *S*. Typhimurium to consume microbiota-derived 1,2-propanediol in the inflamed gut, thereby driving a luminal pathogen expansion [20]. We analyzed the fitness of *S*. Typhimurium mutants in gnotobiotic or conventional mice to test this prediction.

## Results

### 1,2-propanediol-utilization confers a growth advantage during inflammation

**Fig 1:**
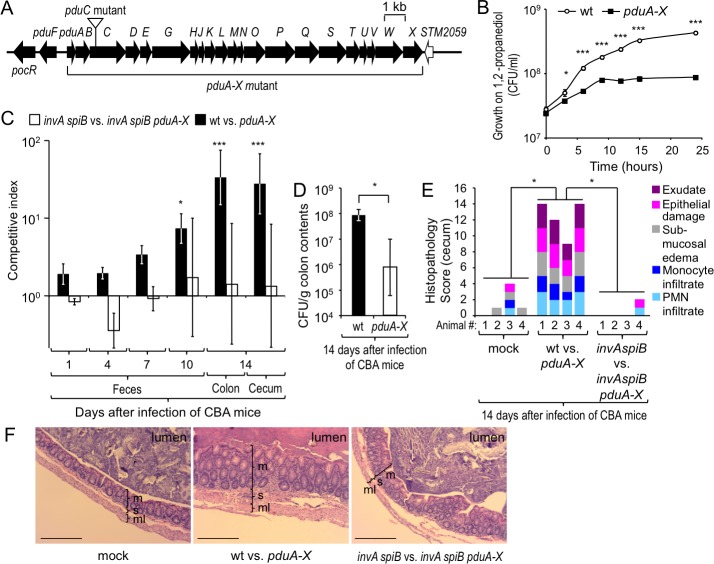
1,2-propanediol utilization confers an advantage during growth in the inflamed murine intestine. (A) Genetic organization of the *pdu* operon. (B) Anaerobic growth of *S*. Typhimurium wild type and the *pduA-X* mutant in minimal medium supplemented with 5 mM 1,2-propanediol and 40 mM tetrathionate. Data points represent geometric means ± standard error of the mean (s.e.m.) of bacterial numbers (CFU/ml) recovered at the indicated time points. (C and E) CBA mice were mock infected (mock) or infected intragastrically with a 1:1 mixture of the indicated *S*. Typhimurium strains. *N* is indicated in panel E. (C) Bars represent geometric means ± s.e.m. of the competitive indices. (D) CBA mice were infected intragastrically with 10^9^ CFU of either the *S*. Typhimurium wild type (wt) or a *pduA-X* mutant. *N* is indicated in S2E Fig. Bars represent geometric means ± s.e.m. of bacteria recovered from colon contents (CFU/g). (E) Histopathological changes were scored in sections of the cecum. Each bar represents the combined scoring results for one individual animal. (F) Representative images of histological sections from the cecum. Scale bar represents 100 μm. M, mucosa; s, submucosa; ml, muscularis; * *P* < 0.05, *** *P* < 0.001.

To determine whether 1,2-propanediol-utilization confers a benefit in the environment of the large intestine, we constructed a *S*. Typhimurium strain lacking the *pduABCDEGHJKLMNOPQSTUVWX* gene cluster (*pduA-X* mutant) (Fig 1A). Compared to the *S*. Typhimurium wild type strain the *pduA-X* mutant grew poorly in minimal medium containing 1,2-propanediol as carbon source (Fig 1B), but showed no growth defect in rich medium (S1A Fig). Genetically resistant (CBA) mice were infected intragastrically with a 1:1 mixture of the *S*. Typhimurium wild type (IR715) and a *pduA-X* mutant (FF128) to compare the fitness of both strains. By 14 days after infection, the *S*. Typhimurium wild type was recovered in significantly higher numbers from cecal and colon contents than a *pduA-X* mutant (*P* < 0.01), suggesting that 1,2-propanediol utilization conferred a benefit for growth of the pathogen in the large intestine (Figs 1C and S2A). Similar results were obtained with a *S*. Typhimurium mutant carrying an insertion in the *pduC* gene (S1B and S2B Figs). After bypassing the gut by the intraperitoneal route of inoculation, the *S*. Typhimurium wild type was recovered in similar numbers as a *pduA-X* mutant from organs of mice infected with a 1:1 mixture of both strains (S2C Fig). Growth of the *pduA-X* mutant in genetically resistant mice could be restored by re-introducing the intact *pdu* operon through transduction (FF484) (S2D Fig).

Next, we infected mice with either the *S*. Typhimurium wild type or a *pduA-X* mutant. By 14 days, mice infected with the *S*. Typhimurium wild type carried a significantly higher pathogen burden in colon contents than mice infected with a *pduA-X* mutant (*P* < 0.05) (Fig 1D), although no significant differences in the severity of intestinal pathology between both groups were noted (S2E Fig).

Since genetically resistant mice develop severe intestinal inflammation by approximately 10 days after infection [21], we wanted to investigate whether the fitness advantage conferred by 1,2-propanediol utilization genes required severe colitis induced by virulence factors. To this end, T3SS-1 and T3SS-2 were inactivated using mutations in the *invA* and *spiB* genes, respectively. When mice (CBA) were infected intragastrically with a 1:1 mixture of an *invA spiB* mutant (FF183) and a *invA spiB pduA-X* mutant (FF383), both strains were recovered in similar numbers from cecal and colon contents 14 days after infection (Fig 1C). Mice infected with virulent *S*. Typhimurium strains (i.e. a mixture of wild type and *pduA-X* mutant) developed severe acute inflammation in the cecal mucosa, while no marked inflammatory changes were observed in mice infected with avirulent *S*. Typhimurium strains (i.e. a mixture of *invA spiB* mutant and *invA spiB pduA-X* mutant) (Fig 1E and 1F). These data suggested that the utilization of 1,2-propanediol conferred a fitness advantage upon *S*. Typhimurium during growth in the inflamed intestine.

### Tetrathionate respiration is dispensable for 1,2-propanediol-utilization *in vivo*

**Fig 2:**
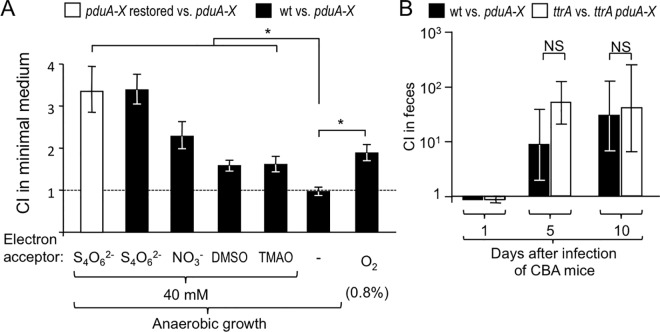
Tetrathionate respiration supports growth of *S*. Typhimurium on 1,2-propanediol *in vitro*, but is not essential for 1,2-propanediol utilization *in vivo*. (A) Competitive growth (*N* = 5) of the indicated *S*. Typhimurium strains on 1,2-propanediol under microaerobic conditions (0.8% oxygen) or anaerobically in the presence of tetrathionate (S_4_O_6_^2-^), nitrate (NO_3_^-^), dimethyl sulfoxide (DMSO) or trimethylamine *N*-oxide (TMAO). Bars represent geometric means ± s.e.m. of the competitive index (CI) recovered after 24 hours. (B) CBA/J mice (*N* = 4) were infected with a 1:1 mixture of the indicated *S*. Typhimurium strains. Bars represent geometric means ± s.e.m. of the CI. * *P* < 0.05, NS not statistically significantly different; wt, *S*. Typhimurium wild type; *pduA-X*, *pduA-X* mutant; *pduA-X* restored, the intact *pdu* operon was introduced into the *pduA-X* mutant by transduction; *ttrA*, *ttrA* mutant; *ttrA pduA-X*, *ttrA pduA-X* mutant.

A variety of exogenous electron acceptors can be generated during intestinal inflammation, including tetrathionate, nitrate, organic *S-*oxides (such as DMSO) or organic *N-*oxides (such as TMAO), which support growth by anaerobic respiration (summarized in [22]). We next investigated whether exogenous electron acceptors would support anaerobic growth of *S*. Typhimurium on 1,2-propanediol *in vitro*. To this end, minimal medium containing 1,2-propanediol as the sole carbon source was supplemented with tetrathionate, nitrate, dimethyl sulfoxide (DMSO) or trimethylamine *N*-oxide (TMAO) and inoculated with a 1:1 mixture of the *S*. Typhimurium wild type and a *pduA-X* mutant. The ability to utilize 1,2-propanediol conferred the largest fitness advantage in media supplemented with tetrathionate, followed by media supplemented with nitrate, DMSO and TMAO (Fig 2A). In contrast, 1,2-propanediol-utilization did not confer a growth advantage in the absence of exogenous electron acceptors, which was consistent with a previous report [19]. Growth of the *pduA-X* mutant on 1,2-propanediol in media supplemented with tetrathionate could be restored by re-introducing the intact *pdu* operon through transduction (FF484).

Since tetrathionate becomes available during inflammation [6], it has been proposed that this electron acceptor might enable *S*. Typhimurium to utilize 1,2-propanediol during colitis [20]. To test this prediction, we inactivated the *ttrA* gene, encoding tetrathionate reductase and infected genetically resistant (CBA) mice intragastrically with a 1:1 mixture of a *S*. Typhimurium *ttrA* mutant (SW661) and a *ttrA pduA-X* mutant (PT305). Surprisingly, 1,2-propanediol-utilization conferred a fitness advantage even after genetic ablation of tetrathionate respiration by a mutation in *ttrA* (Fig 2B). These data refuted the hypothesis that tetrathionate respiration was necessary for the growth benefit conferred by 1,2-propanediol-utilization.

### Anaerobic respiration is not the only driver of 1,2-propanediol-utilization

**Fig 3:**
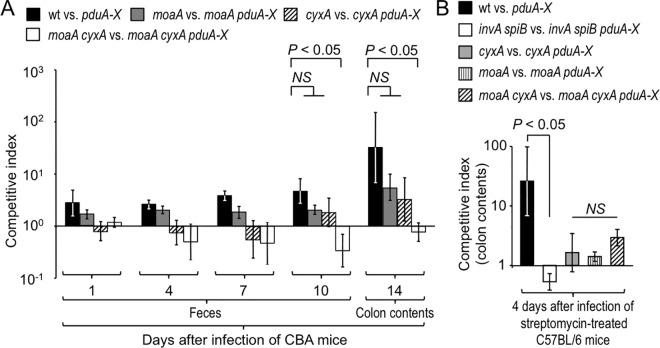
Aerobic and anaerobic respiration cooperates to support growth of *S*. Typhimurium on 1,2-propanediol in the inflamed intestine. CBA mice (A) or streptomycin treated C57BL/6 mice (B) were orally infected with a 1:1 mixture of the indicated *S*. Typhimurium strains. Bars represent geometric means ± s.e.m. of the competitive indices determined at the indicated time points. (A) wt vs. *pduA-X*, *N* = 4; *moaA* vs. *moaA pduA-X*, *N* = 9; *cyxA* vs. *cyxA pduA-X*, *N* = 9; *moaA cyxA* vs. *moaA cyxA pduA-X*, *N* = 10. (B) wt vs. *pduA-X*, *N* = 4; *invA spiB* vs. *invA spiB pduA-X*, *N* = 6; *moaA* vs. *moaA pduA-X*, *N* = 5; *cyxA* vs. *cyxA pduA-X*, *N* = 6; *moaA cyxA* vs. *moaA cyxA pduA-X*, *N* = 5.

The enzymes that enable *S*. Typhimurium to use tetrathionate, nitrate, organic *S-*oxides (such as DMSO) or organic *N-*oxides (such as TMAO) as respiratory electron acceptors under anaerobic conditions contain molybdopterin, a cofactor also required for the use of formate as an electron donor (reviewed in [22]). To determine whether anaerobic respiration contributed to 1,2-propanediol-utilization, we inactivated the *S*. Typhimurium *moaA* gene, encoding the enzyme catalyzing the first step in the molybdopterin cofactor biosynthesis. Genetically resistant (CBA) mice were inoculated intragastrically with a 1:1 mixture of the *S*. Typhimurium wild type and a *pduA-X* mutant or with a 1:1 mixture of a *moaA* mutant (FF294) and a *moaA pduA-X* mutant (FF284). Interestingly, mutational inactivation of *moaA* did not abrogate the fitness advantage conferred by 1,2-propanediol-utilization (Fig 3A). These data suggested that exogenous electron acceptors for anaerobic respiration were not solely responsible for the ability of *S*. Typhimurium to utilize 1,2-propanediol during colitis.

### Aerobic and anaerobic respiration contribute to 1,2-propanediol-utilization

*S*. Typhimurium-induced colitis is accompanied by increased epithelial oxygenation, driving a cytochrome *bd-*II oxidase-dependent aerobic pathogen expansion by 10 days after infection of genetically resistant (CBA) mice [21]. We therefore deleted *cyxA*, the gene encoding cytochrome *bd-*II oxidase, to investigate whether aerobic respiration contributed to 1,2-propanediol-utilization. Genetically resistant (CBA) mice were inoculated intragastrically with a 1:1 mixture of a *cyxA* mutant (FF286) and a *cyxA pduA-X* mutant (FF288). Deletion of *cyxA* did not abrogate the fitness advantage conferred by 1,2-propanediol-utilization (Fig 3A).

To determine whether 1,2-propanediol-utilization involved cooperation between aerobic and anaerobic respiration, genetically resistant (CBA) mice were infected with a 1:1 mixture of a *cyxA moaA* mutant (FF296) and a *cyxA moaA pduA-X* mutant (FF292). Remarkably, genetic ablation of both aerobic respiration (through inactivation of *cyxA*) and anaerobic respiration (through inactivation of *moaA*) abrogated the fitness advantage conferred by 1,2-propanediol-utilization (*P* 0.05) (Fig 3A).

Next, we studied the role of 1,2-propanediol-utilization in mice that were genetically susceptible to *S*. Typhimurium infection (C57BL/6 mice). Genetically susceptible (C57BL/6) mice become moribund before developing severe acute intestinal inflammation during *S*. Typhimurium infection. However, preconditioning of C57BL/6 mice with streptomycin disrupts the resident microbiota and leads to severe acute cecal inflammation during *S*. Typhimurium infection [23]. Streptomycin-treated C57BL/6 mice were infected intragastrically with a 1:1 mixture of the *S*. Typhimurium wild type and a *pduA-X* mutant or with a 1:1 mixture of an *invA spiB* mutant and a *invA spiB pduA-X* mutant. Four days after infection, 1,2-propanediol-utilization conferred a fitness advantage upon *S*. Typhimurium, as indicated by higher recovery of the wild type than a *pduA-X* mutant (Fig 3B and S3 Fig). In contrast, similar numbers of the *invA spiB* mutant and an *invA spiB pduA-X* mutant were recovered from colon contents. These data provided further support for the idea that benefit conferred by 1,2-propanediol-utilization required the presence of virulence factors.

In streptomycin-treated C57BL/6 mice, inactivation of either *moaA* or *cyxA* reduced the fitness advantage conferred by 1,2-propanediol-utilization (Fig 3B). Collectively, these data provided further support for the idea that *S*. Typhimurium used both aerobic and anaerobic respiration to consume 1,2-propanediol during colitis.

### *Salmonella* requires the gut microbiota to benefit from 1,2-propanediol-utilization

**Fig 4:**
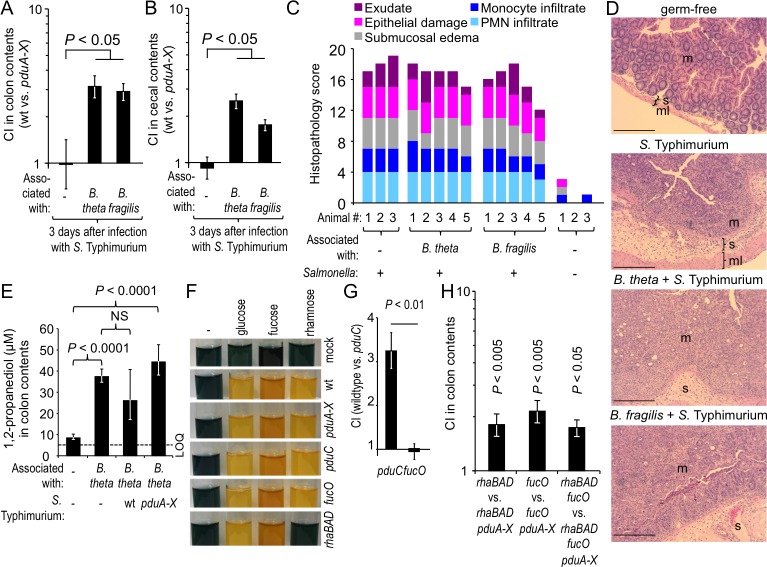
*Salmonella* requires the gut microbiota to benefit from 1,2-propanediol-utilization. (A-B) Germ-free Swiss Webster mice or ex germ-free mice pre-colonized with either *B*. *thetaiotaomicron* (*B*. *theta*) or *B*. *fragilis* for 7 days were infected intragastrically with a 1:1 mixture of *S*. Typhimurium wild type and a *pduA-X* mutant. Bars represent geometric means ± s.e.m. of the competitive index (CI) recovered from colon contents (A) or cecal contents (B). *N* is indicated in panel C. (C) Histopathological changes were scored for sections of the cecum. (D) Representative images of histological sections from the cecum. Scale bar represents 100 μm. m, mucosa; s, submucosa; ml, muscularis. (E) Germ-free Swiss Webster mice or ex germ-free mice mono-associated with *B*. *thetaiotaomicron* for 7 days (*N* = 6) were infected intragastrically with the indicated *S*. Typhimurium strains and the 1,2-propanediol concentration was determined 3 days later. Bars represent geometric means ± s.e.m.; LOQ, limit of quantification. (F) Medium containing the sugars indicated at the top was inoculated with the *S*. Typhimurium strains indicated on the right. Sugar fermentation was detected through the color change (blue to yellow) of a pH indicator. (G) A *pduC* mutant or a *fucO* mutant was grown anaerobically in minimal medium containing fucose as a sole carbon source. The spent culture medium was supplemented with tetrathionate and inoculated with the indicated mixture of *S*. Typhimurium strains to determine the CI after anaerobic growth. (H) Germ-free mice (*N* = 4) were mono-associated with *B*. *thetaiotaomicron* and inoculated with the indicated strain mixtures. (G and H) Bars represent geometric means ± s.e.m. of the competitive index (CI).

Saccharolytic bacteria in the large intestine break down complex carbohydrates, thereby liberating monosaccharides, such as fucose and rhamnose, which can be fermented to generate 1,2-propanediol [11]. To investigate whether utilization of 1,2-propanediol by *S*. Typhimurium required members of the gut-associated microbial community, germ-free (Swiss-Webster) mice were infected with a 1:1 mixture of the *S*. Typhimurium wild type and a *pduA-X* mutant. Equal recovery of both strains from colon contents suggested that in the absence of the gut microbiota, 1,2-propanediol-utilization did not confer a luminal growth advantage in the mouse intestine (Fig 4A and 4B). When mice mono-associated with *Bacteroides fragilis* or *Bacteroides thetaiotaomicron* were infected with a 1:1 mixture of the *S*. Typhimurium wild type and a *pduA-X* mutant, 1,2-propanediol-utilization conferred a fitness advantage (Fig 4A and 4B), which was not due to changes in intestinal inflammation between the groups (Fig 4C and 4D), suggesting that *S*. Typhimurium required the presence of saccharolytic bacteria to utilize 1,2-propanediol in the gut.

Next, we determined whether *B*. *thetaiotaomicron* was a source of 1,2-propanediol in the large intestine. To this end, germ-free mice were mock-inoculated or mono-associated with *B*. *thetaiotaomicron* and the concentration of 1,2-propanediol in colon contents was determined by gas chromatography/mass spectrometry (GC/MS). In mock-inoculated germ-free mice, the concentration of 1,2-propanediol was close to the limit of quantification. In contrast, 1,2-propanediol was present at a concentration of approximately 40 μM in *B*. *thetaiotaomicron* mono-associated mice (Fig 4E). These data suggested that *B*. *thetaiotaomicron* is a source of 1,2-propanediol in the large intestine. To investigate whether consumption of 1,2-propanediol by *S*. Typhimurium would reduce the luminal concentration of this metabolite, mice mono-associated with *B*. *thetaiotaomicron* were infected with the *S*. Typhimurium wild type or a *pduA-X* mutant. While there was a trend that the luminal 1,2-propanediol concentrations were lower in mice infected with the wild type compared to those infected with the *pduA-X* mutant, this difference did not reach statistical significance (Fig 4E).

Next, we determined whether the ability of the pathogen to generate 1,2-propanediol through fermentation of rhamnose or fucose was necessary for its ability to utilize 1,2-propanediol. To this end, we generated *S*. Typhimurium mutants lacking *rhaBAD* or *fucO*, respectively. A *S*. Typhimurium *rhaBAD* mutant was unable to ferment rhamnose (Fig 4F), but exhibited no growth defect in complex culture medium (S1C Fig). Inactivation of 1,2-propanediol utilization by deleting *pduC* or *pduA-X* did not abrogate the ability of *S*. Typhimurium to ferment rhamnose or fucose (Fig 4F). The *fucO* gene encodes 1,2-propanediol oxidoreductase, the enzyme catalyzing the interconversion of lactaldehyde to 1,2-propanediol [24]. Deletion of *fucO* did not abrogate the ability of *S*. Typhimurium to ferment fucose (Fig 4F) and did not produce a growth defect in complex culture medium (S1C Fig). We next generated spent culture medium by growing a *pduC* mutant or a *fucO* mutant anaerobically in minimal medium containing fucose as a sole carbon source. The spent culture medium generated in this fashion was sterilized, supplemented with tetrathionate and inoculated with a 1:1 mixture of the *S*. Typhimurium wild type and a *pduC* mutant. 1,2 propanediol-utilization did not confer a fitness advantage for anaerobic growth in spent culture medium from a *fucO* mutant (Fig 4G), suggesting that the *fucO* mutant was no longer able to generate 1,2-propanediol as a product of fucose fermentation. We then investigated whether *S*. Typhimurium strains unable to generate 1,2-propanediol from fucose (*fucO* mutant), rhamnose (*rhaBAD* mutant) or either pentose (*fucO rhaBAD* mutant) were still able to use the *pdu* operon for expansion in the large intestine of gnotobiotic mice mono-associated with *B*. *thetaiotaomicron*. The *pdu* operon still conferred a significant fitness advantage upon *S*. Typhimurium strains that could generate 1,2-propanediol neither from fucose nor from rhamnose (Fig 4H), suggesting that 1,2-propanediol generated by *B*. *thetaiotaomicron* (Fig 4E) was sufficient to stimulate growth of the pathogen.

Collectively, these data demonstrated that microbe-derived 1,2 propanediol was a critical carbon source for *S*. Typhimurium, which drove an expansion of the pathogen population during colitis.

## Discussion

Associations revealed by genome and literature mining predict that propanediol utilization pathways are genomic determinants of pathogenicity associated with food poisoning, presumably by promoting growth in the low-oxygen environment of the large intestine [25]. For example, adherent-invasive *Escherichia coli* (AIEC) isolated from intestines of patients with Crohn's disease (CD) contain genes encoding 1,2-propanediol-utilization, while the gene cluster is commonly absent from commensal *E*. *coli* isolates [26]. Comparative genomic analysis of *S*. *enterica* serovars suggest that loci involved in 1,2-propanediol utilization are intact in pathogens causing gastrointestinal disease, such as *S*. *enterica* serovars Typhimurium or Enteritidis, but are often disrupted in closely related pathogens associated exclusively with extraintestinal disease, such as *S*. *enterica* serovars Typhi, Paratyphi A, Gallinarum or Choleraesuis [8–10].

*S*. Typhimurium growth under conditions mimicking the low-oxygen high-osmolarity environment of the gut induces the synthesis of proteins involved in the utilization of 1,2-propanediol [27]. Transcriptional profiling suggests that 1,2-propanediol utilization genes are expressed during *S*. Typhimurium growth in the ceca of chickens [28] and in gnotobiotic mice mono-associated with *B*. *thetatiotaomicron* [29]. Collectively, studies on gene regulation and the *in silico* predictions reviewed above imply that 1,2-propanediol utilization genes might contribute to *S*. Typhimurium growth in the large intestine, but the present report is the first to test this hypothesis using an animal model. Our results provide compelling experimental support for the idea that 1,2-propanediol-utilization contributes to growth of *S*. Typhimurium during gastroenteritis, suggesting that the pathogen thrives during gut inflammation in part by respiring fermentation products generated by other microbes.

While tetrathionate respiration is necessary for growth on ethanolamine *in vivo* [7], efficient 1,2-propanediol-utilization in the mouse intestine required both aerobic and anaerobic respiration. Interestingly, 1,2-propanediol represses transcription of ethanolamine utilization genes to avoid detrimental mixing of shell proteins from the corresponding microcompartments [30]. Thus, the finding that different electron acceptors are required to breakdown 1,2-propanediol and ethanolamine *in vivo* may reflect the need to prevent simultaneous expression of the *pdu* and *eut* gene clusters. In turn, this may restrict utilization of 1,2-propanediol and ethanolamine to microenvironments that differ with regard to electron acceptor availability. However, additional work is needed to test this prediction.

**Fig 5:**
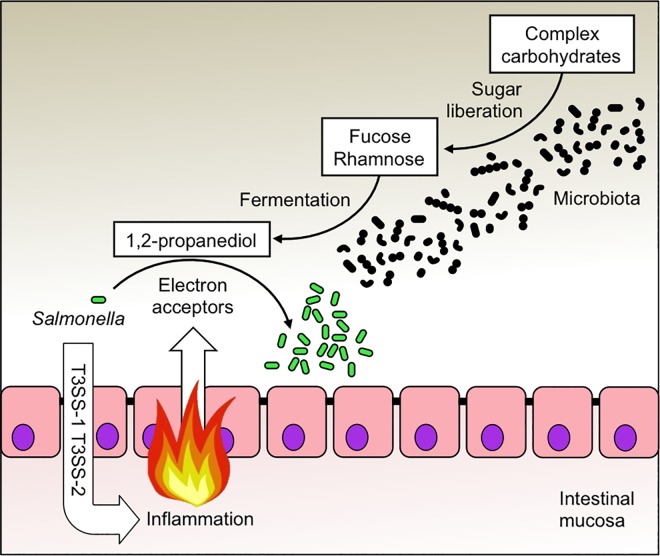
Schematic of *S*. Typhimurium 1,2-propanediol utilization during colitis. The enteric pathogen *S*. Typhimurium (*Salmonella*) uses its virulence factors (T3SS-1 and T3SS-2) to elicit severe acute intestinal inflammation. A side-product of the host inflammatory response is the generation of exogenous electron acceptors, which creates a respiratory nutrient-niche in the lumen of the large intestine. A combination of aerobic and anaerobic respiration enables *S*. Typhimurium to expand in the inflamed intestine by consuming microbe-derived 1,2-propanediol, a metabolite produced during the fermentation of fucose or rhamnose. Monosaccharides, such as fucose and rhamnose, are liberated by the gut microbiota from complex carbohydrates, which escape digestion by host enzymes in the upper intestinal tract and reach the colon.

1,2-propanediol is generated by the gut microbiota through fermentation of methyl-pentoses, such as fucose or rhamnose [11]. Glycoside hydrolases and polysaccharide lyases expressed by the gut microbiota can liberate fucose from complex carbohydrates [29] (Fig 5). Members of the class *Bacteroidia* encode the most diverse array of glycoside hydrolases and polysaccharide lyases, suggesting that this group possesses the largest carbohydrate substrate range within the gut-associated microbial community [31]. Consistent with this idea, 1,2-propanediol-utilization conferred a benefit upon *S*. Typhimurium in mice mono-associated with *B*. *fragilis* or *B*. *thetaiotaomicron*, but not in germ-free mice.

The picture emerging from this and previous work is that respiratory electron acceptors become available during *S*. Typhimurium-induced colitis [6, 21, 32, 33], which enables the pathogen to consume 1,2-propanediol, a process that could be viewed as “dumpster diving” for a fermentation product produced by the gut microbiota [34] (Fig 5). Respiration of 1,2-propanediol allows *S*. Typhimurium to side-step nutritional competition with the fermenting gut microbiota to drive its expansion within the lumen of the large bowel. This outcome is biologically relevant, because an uncontrolled expansion of *S*. Typhimurium during colitis is important for infectious transmission by the fecal oral route [21, 35].

## Materials and Methods

### Ethics statement

This study was performed in strict accordance with the recommendations in the Guide for the Care and Use of Laboratory Animals of the National Institutes of Health and approved by the Institutional Animal Care and Use Committee at the University of California at Davis (protocols #15449, 17140, 17939 and 19235).

### Bacterial strains and plasmids

All strains used in this study are listed in Table 1. *S*. Typhimurium and *E*. *coli* cultures were routinely grown aerobically at 37°C in either Luria-Bertani (LB) broth (10 g/l tryptone, 5 g/l yeast extract, 10 g/l NaCl) or on LB agar plates (1.5% Difco agar) unless indicated otherwise. When necessary, antibiotics were added to the medium at the following concentrations: Nalidixic acid (Nal) 50 mg/l, Kanamycin (Km) 100 mg/l, Chloramphenicol (Cm) 30 mg/l, Carbenicillin (Carb) 100 mg/L. *B*. *thetaiotaomicron* and *B*. *fragilis* were routinely grown inside an anaerobe chamber (Bactron I Anaerobic Chamber; Sheldon Manufacturing, Cornelius) at 37°C in either Tryptic Soy Broth (TSB) or on Tryptic Soy agar (TSA) plates (1.5% [w/v] Difco agar) supplemented with 5% (v/v) sheep blood.

**Table 1:** Bacterial strains used in this study.

Designation	Genotype	Reference
***S*. Typhimurium strains**		
IR715	Nalidixic acid-resistant derivative of ATCC 14028s	[40]
FF176	IR715 *phoN*::Tn*10d-*Cam	[41]
SPN487	IR715 *invA spiB*	[41]
FF183	IR715 *phoN*::Tn*10d-*Cam *invA spiB*	[41]
SDL175	IR715 *pduC*::Kan^R^	This study
FF128	IR715 *pduA-X*::Kan^R^	This study
FF383	IR715 *invA spiB pduA-X*::Kan^R^	This study
SW661	IR715 *ttrA*::pSW171	[6]
PT305	IR715 *ttrA*::pSW171 *pduA-X*::Kan^R^	This study
FF283	IR715 Δ*moaA*	This study
FR102	IR715 *cyxA*::pPT48	[21]
FF294	IR715 *phoN*::Tn*10d-*Cam Δ*moaA*	This study
FF284	IR715 *moaA pduA-X*::Kan^R^	This study
FF286	IR715 *phoN*::Tn*10d-*Cam *cyxA*::pPT48	This study
FF288	IR715 *cyxA*::pPT48 *pduA-X*::Kan^R^	This study
FF296	IR715 *phoN*::Tn*10d-*Cam *moaA cyxA*::pPT48	This study
FF292	IR715 *moaA cyxA*::pPT48 *pduA-X*::Kan^R^	This study
FF489	IR715 *rhaBAD*	This study
FF495	IR715 *rhaBAD pduA-X*::Kan^R^	This study
FF499	IR715 *phoN*::Tn*10d-*Cam *rhaBAD*	This study
PT206	IR715 *fucO*::pPT21	This study
FF509	IR715 *phoN*::Tn*10d-Cam fucO*::pPT21	This study
FF497	IR715 *fucO*::pPT21 *pduA-X*::Kan^R^	This study
FF505	IR715 *phoN*::Tn*10d-Cam rhaBAD fucO*::pPT21	This study
FF503	IR715 *rhaBAD fucO*::pPT21 *pduA-X*::Kan^R^	This study
FF480	IR715 *pduP*::pRDH10	This study
FF484	FF128 restored to wild type	This study
***Bacteroides* strains:**		
*B*. *thetaiotaomicron* VPI-5482	Isolate from human feces	ATCC 29148
*B*. *fragilis*	Isolate from appendix abscess	ATCC25285
***E*. *coli* strains:**		
DH5α λ*pir*	F^-^ *endA1 hsdR17* (r^-^m^+^) *supE44 thi-1 recA1 gyrA relA1* Δ*(lacZYA-argF)*_*U189*_ *ϕ80lac* Δ*M15* λ*pir*	[42]
S17-1 λ*pir*	C600::RP4 2-(Tet::*Mu*) (Kan::Tn7) λ*pir recA1 thi pro hsdR* (r^-^m^+^)	[43]
TOP10	*ϕ80lacZ*Δ*M15 lacX74 recA1 araD139* Δ*(ara-leu) 7697 galU galK rpsL endA1 nupG*	Invitrogen

### Construction of *S*. Typhimurium mutants

All strains, plasmids and primers used in this study are listed in Tables 1–3. Standard cloning techniques were used to generate the plasmids used in this study. PCR products were confirmed by sequencing (SeqWright Fisher Scientific, Houston). Suicide plasmids were propagated in *E*. *coli* DH5α λ*pir*. Phage P22 HT *int-105* was used for generalized transduction. Transductants were cleaned from phage contamination on Evans blue-Uranine (EBU) plates and tested for phage sensitivity by cross-streaking against P22 H5.

**Table 2:** Plasmids used in this study.

Designation	Relevant characteristic	Reference
pCR2.1	Cloning vector	Invitrogen
pGP704	*ori*(R6K) *mobRP4* Carb^R^	[44]
pRDH10	*ori*(R6K) *mobRP4 sacRB* Tet^R^ Cm^R^	[45]
pBS34	pBluescript II KS+, KSAC cassette, Carb^R^, Km^R^	[46]
pPT33	pCR2.1 with region upstream of *pduA*	This study
pPT34	pCR2.1 with region downstream of *pduX*	This study
pPT35	pGP704 with region upstream of *pduA*	This study
pPT36	pGP704 with regions upstream of *pduA* and downstream of *pduX*	This study
pPT37	pGP704 with Kan^R^ cassette inserted between p*duA* and *pduX fragments*	This study
pPT48	pGP704 carrying an internal fragment of *cyxA* (annotated as STM14_0423 in ATCC 14028s)	[21]
pCAL25	pRDH10 carrying up-/downstream regions of *moaA*	This study
pRhaBAD	pRDH10 carrying flanking regions of *rhaBAD*	This study
pPT20	pCR2.1 carrying an internal fragment of *fucO*	This study
pPT21	pGP704 carrying an internal fragment of *fucO*	This study
pCR-pduP	pCR2.1 carrying an internal fragment of *pduP*	This study
pRDH10-pduP	pRDH10 carrying an internal fragment of *pduP*	This study
pKD4	Carries the Kan^R^ inserted in *pduC*	[37]
pKD46	Carries λ Red recombinase under an arabinose inducible promoter	[37]

**Table 3:** Primers used in this study.

Primer	Sequence in 5’ - 3’
*Deletion of pduA-X operon*	
pduA-P1	TCTAGAAGACCTCGCATGGAG
pduA-P2	GTCGACCATTGCGCCTTTAG
pduX-P3	TCTAGAAGCCTGATATACGC
pduX-P4	CCCGGGCTAACGATCGCCCAC
Deletion of *moaA*	
moaA-P1	ATCTTAGTCGACGGCAGACTCAGTTCG
moaA-P2	GACGAGTCTAGAGAGTGTTTACTGTGG
moaA-P3	TAAGAATCTAGAGCGGCGAAGAAGACG
moaA-P4	CCAGTTGTCGACTCAGTTCCTGTAAGC
Deletion of *pduC*	
STM20405'+P1	CGATCGTCCGTCCTACATCTGATACCCACGAGGCTGATTCGTGTAGGCTGGAGCTGCTTC
STM20403'+P2	CTGGCGCAGCAATTTTTCATTAATTTCCATTTCTCACCCCCATATGAATATCCTCCTTAG
STM2040 5'	CTCGGTTCTGAACCGAAAAA
STM2040 3'	CGGAGTACGTCTTCAATTAT
*Deletion of rhaBAD operon*	
rhaBAD-P1	TCATGGCGACCACACCCGTCCTGTGTCAAACCGAAGGTTTCATC
rhaBAD -P2	TGCCCGATTATTTCGAATACGCCGTTTATCG
rhaBAD -P3	AACGGCGTATTCGAAATAATCGGGCATCAGCAG
rhaBAD -P4	CGGCCACGATGCGTCCGGCGTAGAGAGCAAACACCGTACCCTG
*Deletion of fucO operon*	
fucO-P1	GGTACCAGCGCTGATTGTTACCG
fucO-P2	GATATCGCATGGGTTAGCGC
Restoration of FF128 (*pduA-X*::Kan^R^)	
pduP-P1	GAATTCGCGTTATCGGTTCGG
pduP-P2	GAATTCCTTTCACCAGGTCCGC

To construct the *pduA-X* mutant (FF128), a region upstream and downstream of *pduA and pduX*, respectively, were PCR amplified from the *S*. Typhimurium wild-type strain IR715. Both PCR products (the flanking regions of *pduA* and *pduX* genes) were subcloned into pCR2.1 to obtain pPT33 and pPT34, respectively. The inserted PCR fragments were confirmed by sequencing. The *pduA* fragment from pPT33 was cloned into the suicide plasmid pGP704 by using SalI and XbaI restriction sites to obtain pPT35. The *pduX* fragment from pPT34 was digested by XbaI and SmaI and then inserted into pPT35 to yield pPT36. Next, the Kan^r^ cassette from pBS34 was inserted into pPT36 to obtain pPT37. Plasmid pPT37 was transformed into *E*. *coli* S17-1λ*pir*. Plasmid pPT37 was conjugated into the *S*. Typhimurium wild type (IR715) and an *invA spiB* mutant (SPN487) using *E*. *coli* S17-1λ*pir* as a donor strain to generate FF128 (IR715 *pduA-X*::Kan^R^) and FF383 (IR715 *invA spiB pduA-X*::Kan^R^), respectively. Exconjugants were plated onto LB+Nal+Kan and tested for Carb^S^ to select for clones that carried the deletion.

To construct the *moaA* mutant, a region upstream and downstream of *moaA* were PCR amplified from the *S*. Typhimurium wild-type strain IR715 introducing XbaI and SalI restriction sites into both fragments. Then, both PCR products were digested with XbaI ligated together and cloned into pRDH10 using SalI restriction sites yielding plasmid pCAL25. The inserted PCR fragments were confirmed by sequencing. Plasmid pCAL25 was conjugated into *S*. Typhimurium IR715 (wild-type) and FF176 (*phoN*::Tn10d-Cam) using *E*. *coli* S17-1λ*pir* as a donor strain to generate FF283 (IR715 *moaA*) and FF294 (*phoN*::Tn10d-Cam *moaA*), respectively. Exconjugants were plated onto LB+Nal+Cm to select for clones that had integrated the suicide plasmid. Sucrose counter-selection was performed as published previously [36]. Strains that were sucrose resistant and Cm^S^ were verified by PCR.

The *pduC* mutant was constructed using the λ Red recombinase method as previously described [37]. PCR products were generated using primers STM20405’+P1 and STM20403’+S2 with pKD4 as the DNA template [37]. The appropriate length of the PCR products, approximately 1.6 kb, was ensured by agarose gel electrophoresis. The PCR product was electroporated into electrocompetent ATCC14028 carrying pKD46, a temperature sensitive plasmid which carries λ Red recombinase under an arabinose inducible promoter [37]. The recipient was prepared as previously described [37]. Following electroporation, transformants were allowed to recover for 1 hour at 37°C and were then plated onto LB+Kan and grown at 37°C to select for clones that had integrated the PCR product. Insertion of the Kan^R^ was confirmed by PCR using primers STM2040 5’ with previously described primer k1 and STM2040 3’ with primer k2 [37]. The mutation was also confirmed by Southern blot using a digoxigenin-labeled probe generated by PCR using primers k1 and kt with pKD4 DNA as template. A P22 lysate of the resultant strain was used to transduce the *pduC*::Kan^R^ into IR715 to generate SDL175.

To construct the *rhaBAD* mutant, regions flanking the *rhaBAD* operon were PCR amplified from the *S*. Typhimurium wild-type strain IR715 and cloned into BamHI-digested pRDH10 using Gibson Assembly Master Mix (NEB) yielding plasmid pRhaBAD. Plasmid pRhaBAD was conjugated into *S*. Typhimurium IR715 (wild-type) and FF128 (*pduA-X*::Kan^R^) using *E*. *coli* S17-1λ*pir* as a donor strain to generate FF489 (IR715 *rhaBAD*) and FF495 (*rhaBAD pduA-X*::Kan^R^), respectively. Exconjugants were plated onto LB+Nal+Cm to select for clones that had integrated the suicide plasmid. Sucrose counter-selection was performed as published previously [36]. Strains that were sucrose resistant and Cm^S^ were verified by PCR.

To construct the *fucO* mutant, an internal fragment of the *fucO* gene was PCR amplified from the *S*. Typhimurium wild-type strain IR715 and cloned into pCR2.1 to generate pPT20. The *fucO* fragment from pPT20 was cloned into suicide plasmid pGP704 to obtain pPT21. Using *E*. *coli* S17-1λ*pir* as a donor strain, plasmid pPT21 was conjugated into *S*. Typhimurium wild type (IR715), the *phoN*::Tn*10d-Cam rhaBAD* mutant (FF499) and the *rhaBAD pduA-X*::Kan^R^ mutant (FF495) to generate PT206 (IR715 *fucO*::pPT21), FF505 (IR715 *phoN*::Tn*10d-Cam rhaBAD fucO*::pPT21) and FF503 (IR715 *rhaBAD fucO*::pPT21 *pduA-X*::Kan^R^), respectively. Exconjugants were plated onto LB+Nal+Carb to select for clones that carried the plasmid insertion.

A P22 lysate of strain FF128 was used to transduce the *pduA-X*::Kan^R^ mutation into SW661 (*ttrA*), FF283 (*moaA*), FR102 (*cyxA*::pPT48) and PT206 (*fucO*::pPT21) to obtain strains PT305 (*ttrA*::pSW171 *pduA-X*::Kan^R^) FF284 (*moaA pduA-X*::Kan^R^), FF288 (*cyxA*::pPT48 *pduA-X*::Kan^R^) and FF497 (*fucO*::pPT21 *pduA-X*::Kan^R^), respectively.

A P22 lysate of strain FR102 was used to transduce the *cyxA*::pPT48 mutation into FF176 (*phoN*::Tn*10d-*Cam), FF294 *phoN*::Tn*10d-*Cam *moaA*) and FF284 (*moaA pduA-X*::Kan^R^) to generate strains FF286 (*phoN*::Tn*10d-*Cam *cyxA*::pPT48), FF296 (*phoN*::Tn*10d-*Cam *moaA cyxA*::pPT48) and FF292 (IR715 *moaA cyxA*::pPT48 *pduA-X*::Kan^R)^, respectively.

A P22 lysate of strain FF176 was used to transduce the *phoN*::Tn*10d-*Cam mutation into FF283 (IR715 *moaA*), FF489 (*rhaBAD*) and PT206 (*fucO*::pPT21) yielding strains FF294 (IR715 *phoN*::Tn*10d-*Cam *moaA*), FF499 (*phoN*::Tn*10d-*Cam *rhaBAD*) and FF509 (*phoN*::Tn*10d-*Cam *fucO*::pPT21), respectively.

To restore the *pduA-X*::Kan^R^ mutant (FF128), an internal fragment of the *pduP* gene (part of the *pduA-X* operon) was PCR amplified from the *S*. Typhimurium wild-type strain IR715 and cloned into pCR2.1 to generate pCR-*pduP*. The *pduP* fragment from pCR2.1 was cloned into suicide plasmid pRDH10 to obtain pRDH10-pduP. Using *E*. *coli* S17-1λ*pir* as a donor strain, plasmid pRDH10-pduP was conjugated into *S*. Typhimurium wild type (IR715) yielding strain FF480. A P22 lysate of strain FF480 was used to transduce *pduP*::pRDH10 into FF128. Sucrose counter-selection was performed as published previously [36]. Strains that were sucrose resistant and Cm^S^ were verified by PCR.

### *In vitro* growth assays

#### Anaerobic growth assays

Growth assays were performed in No-carbon-E (NCE) medium [19] supplemented with trace metals (0.3 μM CaCl_2_, 0.1 μM ZnSO_4_, 0.045 μM FeSO_4_, 0.2 μM Na_2_Se_2_O_3_, 0.2 μM Na_2_MoO_4_, 2 μM MnSO_4_, 0.1 μM CuSO_4_, 3 μM CoCl_2_, 0.1 μM NiSO_4_) and 5 mM 1,2-propanediol (Sigma-Aldrich) as the sole carbon source. For anaerobic growth, cultures were incubated inside an anaerobe chamber with one of the following electron acceptors added to the medium: 40 mM sodium tetrathionate, 40 mM sodium nitrate, 40 mM dimethyl sulfoxide (DMSO) or 40 mM trimethylamine *N*-oxide (TMAO) (all purchased from Sigma-Aldrich). For microaerobic growth cultures were incubated inside a hypoxia chamber set at 0.8% oxygen. To enhance initial growth, media were inoculated with a single strain or with an equal mixture of indicated *S*. Typhimurium strains resuspended in LB and incubated at 37°C for 24 hours, which was based on a similar protocol [38]. Bacterial numbers were determined by plating serial ten-fold dilutions onto LB agar containing the appropriate antibiotics. The ratios of recovered wild-type and mutant bacteria after 24 hours were normalized to the ratio at 0 hours to calculate the competitive index.

#### Sugar fermentation assay

5 ml of fermentation broth (peptone, 10 g/l; Bromothymol blue, 0.024 g/l; final pH 7.4±0.1) supplemented with the indicated carbon source (galactarate, glucarate, galactose, glucose, mannose or rhamnose, 10g/L each) or the control broth (no sugar added) were inoculated with 10 μl of an over night culture of each indicated *S*. Typhimurium strain and incubated statically at 37°C for 24 hours. Fermentation of the sugar in the broth is indicated by a color change from blue to yellow.

### Animal experiments

All animal experiments were approved by the Institutional Animal Care and Use Committees at the University of California, Davis. Female C57BL/6J and CBA/J wild-type mice aged 8–10 weeks were obtained from The Jackson Laboratory (Bar Harbor). Germ-free Swiss-Webster mice were bred in house.

C57BL/6 Mice were treated with an oral dose of 20 mg streptomycin 24 hours before oral infection with 0.1 ml LB broth (mock-infected) or with 1 x 10^9^ CFU of a 1:1 mixture of the indicated *S*. Typhimurium strains. Mice were euthanized 4 days after infection, cecal and colon contents were collected for enumeration of bacterial numbers and the cecal tip was collected for histopathology scoring. Bacterial numbers were determined by plating serial ten-fold dilutions onto LB agar containing the appropriate antibiotics.

For intraperitoneal infection, C57BL/6 mice were injected with 10^5^ CFU of a 1:1 mixture of the indicated *S*. Typhimurium strains. Mice were euthanized 2 days after infection and spleen, liver and mesenteric lymph nodes were collected for enumeration of bacterial numbers. Tissues were homogenized and bacterial numbers were determined by plating serial ten-fold dilutions onto LB agar containing the appropriate antibiotics.

CBA mice were infected with either 0.1 ml of LB broth (mock-infected) or *S*. Typhimurium in LB broth. For single infections, mice were inoculated with 1 x 10^9^ CFU of the indicated *S*. Typhimurium strains. For competitive infections, mice were inoculated with 1 x 10^9^ CFU of a 1:1 mixture of the indicated strains. Fecal pellets were collected at the indicated time points to monitor colonization over time. Mice were euthanized at 14 days after infection, cecal and colon contents were collected for enumeration of bacterial numbers and the cecal tip was collected for histopathology scoring. Bacterial numbers were determined by plating serial ten-fold dilutions onto LB agar containing the appropriate antibiotics.

Germ-free Swiss Webster mice were obtained from Taconic Farms. Mice were bred and housed under germ-free conditions inside gnotobiotic isolators (Park Bioservices, LLC). Weekly cultures were performed to monitor the germ-free status of the mice. For experiments, male and female 6–8 weeks old mice were transferred to a biosafety cabinet and maintained in sterile cages for the duration of the experiment. Each recipient germ-free mouse was orally inoculated with TSB (mock-infected) or the indicated *Bacteroides* strain resuspended in TSB. Mice were mono-colonized for 7 days with either *B*. *thetaiotaomicron* or *B*. *fragilis* before oral challenge with an equal mixture of 10^9^ CFU of the indicated *S*. Typhimurium strains. Mice were euthanized 3 days after challenge with *S*. Typhimurium, cecal and colon contents were collected for enumeration of bacterial numbers and the cecal tip was collected for histopathology scoring. Bacterial numbers were determined by plating serial ten-fold dilutions onto LB agar containing the appropriate antibiotics.

When mice were infected with a 1:1 mixture of bacterial strains, the ratio of recovered bacterial strains was normalized to the ratio present in the inoculum to calculate the competitive index.

### Histopathology

Cecal tissue was fixed in 10% phosphate- buffered formalin and 5 μm sections of the tissue were stained with hematoxylin and eosin. Blinded scoring of tissue sections was performed by a veterinary pathologist based on the criteria listed in S4 Fig. Representative images were taken using an Olympus BX41 microscope.

### 1,2-propanediol measurements

Germ-free mice were orally inoculated with TSB (mock-infected) or *B*. *thetaiotaomicron* and colonized for 7 days before oral challenge with LB (mock-infected) or 10^8^ CFU of *S*. Typhimurium wild-type or the *pduA-X* mutant. Mice were euthanized 3 days after challenge with *S*. Typhimurium and colon contents were collected in 250 μL of sterile PBS spiked with 0.1 mM (final concentration) of deuterated 1,2-propanediol (internal standard). The sample weights were measured for later normalizations to determine the correct concentration. Samples were vortexed for 2 minutes at maximum speed to break up the fecal pellet and create a homogenous solution. Samples were then centrifuged at 6,000 x g, 4°C for 15 minutes, the supernatant was transferred into a new tube and stored at -80°C for further processing. Samples, diluted with acetonitrile at a 1:1 ratio, were incubated for 30 min at room temperature. To remove particles, samples were centrifuged for 15 min at 20,000 g at 4°C. The supernatant was transferred to an autosampler vial for gas chromatography-mass spectrometry analysis. A Gas Chromatograph Mass Spectrometer (Shimadzu TQ-8040 GC/MS/MS) was used with an injection temperature of 250°C, injection split ratio of 5 and an injection volume of 1 μl. The GC oven temperature started at 100°C for 1 min, rising to 250°C at 10°C/min with a final hold at this temperature for 4 min. GC flow rate with helium carrier gas was constant at 35 cm/s. The GC column used was a 30 m × 0.25 mm × 0.25 μm Stabilwax-MS (Restek). The interface temperature was 250°C and ion source temperature was 200°C. The mass spectrometer was set to selected-ion monitoring of the three most abundant *m*/*z*, *m/z* 1 at 29, *m/z* 2 at 43, *m/z* 3 at 45 for 1,2-propanediol and *m/z* 1 at 30, *m/z* 2 at 33 and *m/z* 3 at 49 for the internal standard 1,2-propanediol-d_6_. *m/z* at 45 and *m/z* at 49 were used to quantify 1-2-propanediol and 1,2-propanediol-d_6_ respectively, while the other m/z were used as qualifying ions [39]. Recovery was calculated based on the internal standard. An external standard curve was run in triplicate for quantification.

### Statistical analysis

Student’s *t*-test was performed on logarithmically transformed values for bacterial numbers and competitive indices. A non-parametric test (Man Whitney) was used for comparing histopathology scores.

## Supporting Information

S1 Fig Growth of *S*. Typhimurium strains in rich medium. (A-C) Rich medium (LB broth) was inoculated with one of the indicated *S*. Typhimurium strains and bacterial growth monitored by measuring the optical density at 600 nm (OD600). Each experiment was repeated three times independently.
(PDF)

S2 Fig The *S*. Typhimurium *pduC* mutant is attenuated during growth in the inflamed murine intestine, but not for growth in organs. (A) CBA mice were infected intragastrically with a 1:1 mixture of the indicated *S*. Typhimurium strains. Bars represent geometric means ± s.e.m. of the CFU recovered for each strain at 14 days after infection. (B) CBA mice (*N* = 6) were infected intragastrically with a 1:1 mixture of *S*. Typhimurium wild type and a *pduC* mutant. (C) C57BL/6 mice (*N* = 4) were infected intraperitoneally with a 1:1 mixture of *S*. Typhimurium wild type and a *pduA-X* mutant. (D) CBA mice (*N* = 6) were infected intragastrically with a 1:1 mixture of a *pduA-X* mutant and a strain in which the *pduA-X* mutation had been restored by introducing the intact *pdu* operon through transduction. (A-C) Bars represent geometric means ± s.e.m. of the competitive indices. (E) Histopathological changes were scored in sections of the cecum for the experiment shown in Fig 1D. Each bar represents the combined scoring results for one individual animal. *, *P* < 0.05; **, *P* < 0.01; MLN, mesenteric lymph node.
(PDF)

S3 Fig 1,2-propanediol utilization confers a fitness advantage in C57BL/6 mice. Streptomycin-treated C57BL/6 mice were infected intragastrically with a 1:1 mixture of the indicated *S*. Typhimurium strains. Bars represent geometric means ± s.e.m. of the CFU recovered for each strain at 4 days after infection.
(PDF)

S4 Fig Scoring criteria for the blinded examination of H&E stained cecal tissue sections. Criteria for blinded scoring performed by a veterinary pathologist.
(PDF)
