# Supplementary figures and images for: Respiration of Microbiota-Derived 1,2-propanediol Drives Salmonella Expansion during Colitis
Source: PLoS Pathog. 2017 Jan 5;13(1):e1006129. doi: 10.1371/journal.ppat.1006129 (PMC5215881; doi:10.1371/journal.ppat.1006129)

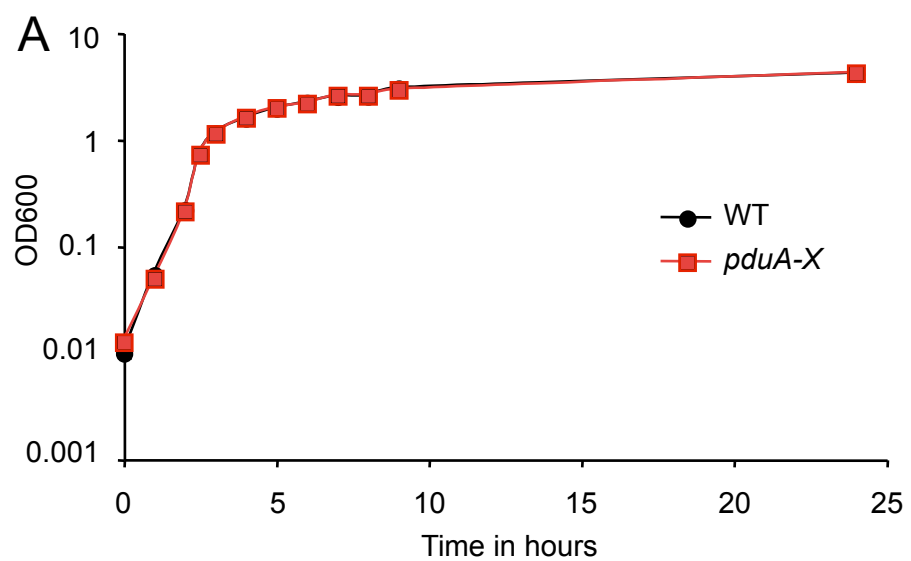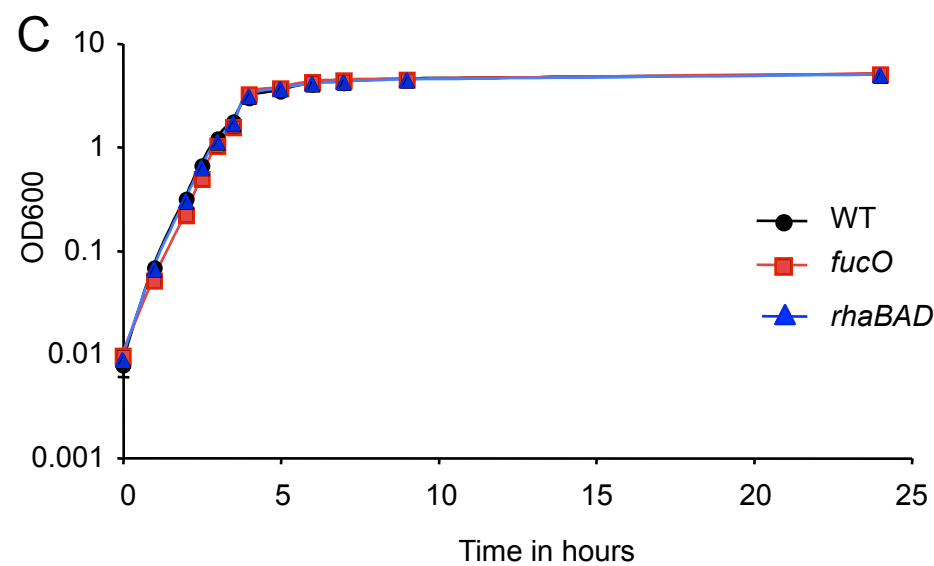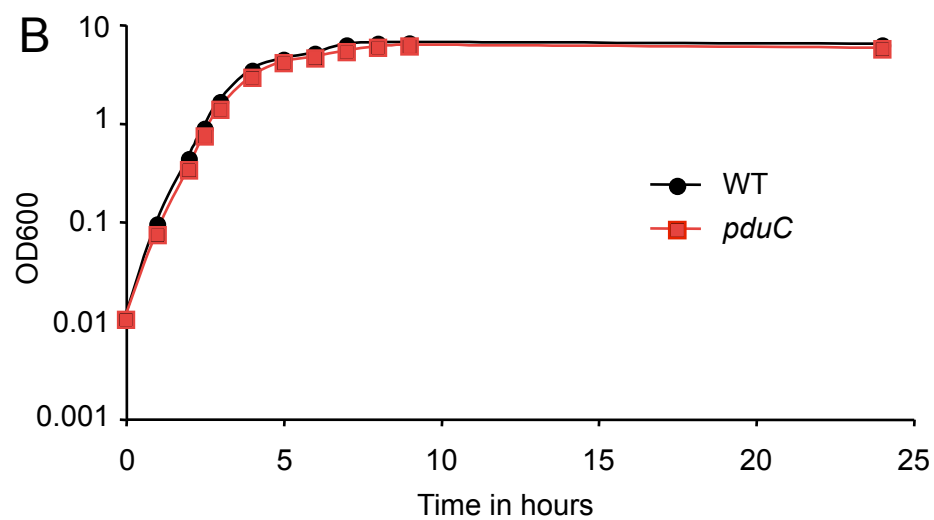

Fig. S1

Supplement: S1 Fig — (A-C) Rich medium (LB broth) was inoculated with one of the indicated S. Typhimurium strains and bacterial growth monitored by measuring the optical density at 600 nm (OD600). Each experiment was repeated three times independently. (PDF) [file ppat.1006129.s001.pdf]

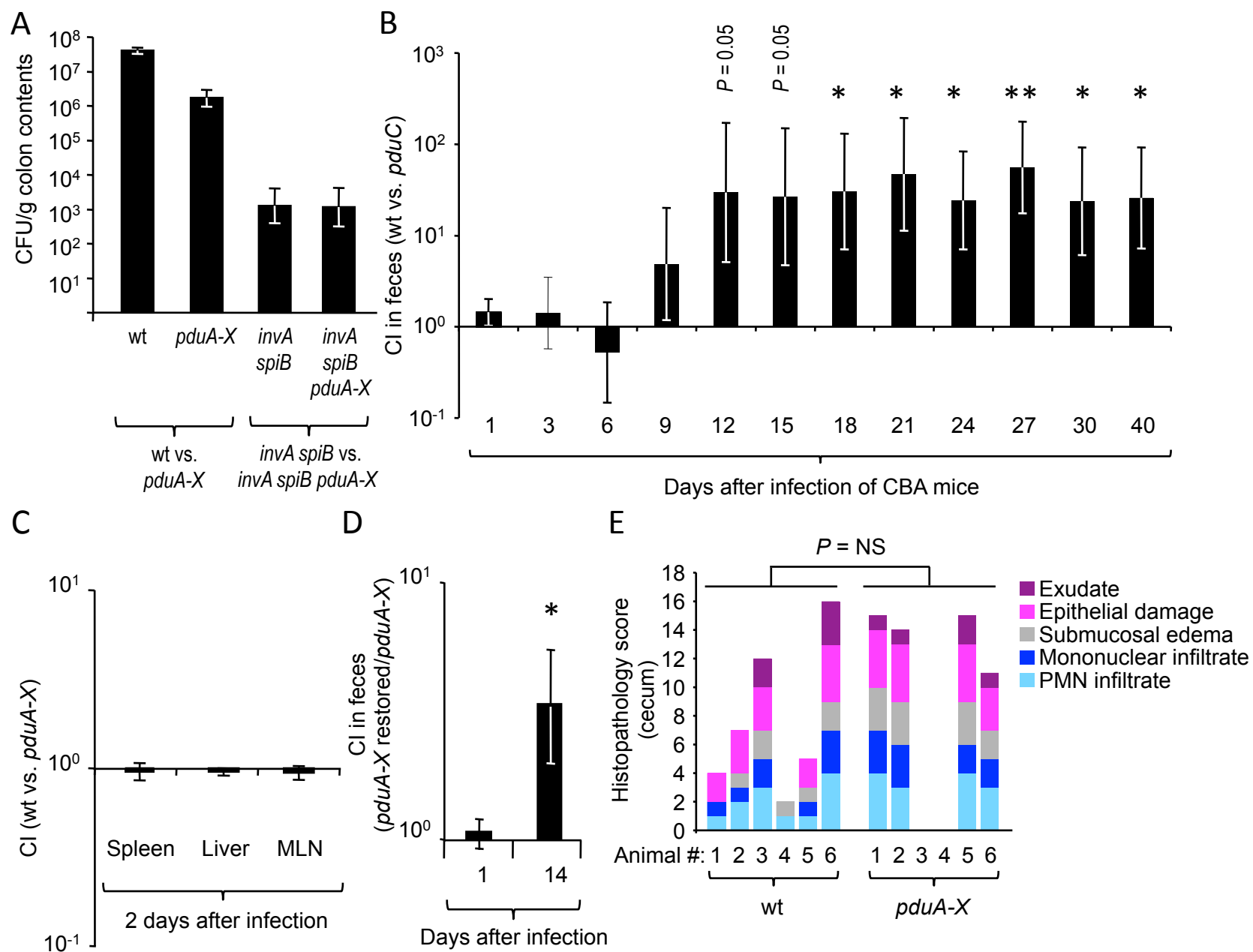

Fig. S2

Supplement: S2 Fig — (A) CBA mice were infected intragastrically with a 1:1 mixture of the indicated S. Typhimurium strains. Bars represent geometric means ± s.e.m. of the CFU recovered for each strain at 14 days after infection. (B) CBA mice (N = 6) were infected intragastrically with a 1:1 mixture of S. Typhimurium wild type and a pduC mutant. (C) C57BL/6 mice (N = 4) were infected intraperitoneally with a 1:1 mixture of S. Typhimurium wild type and a pduA-X mutant. (D) CBA mice (N = 6) were infected intragastrically with a 1:1 mixture of a pduA-X mutant and a strain in which the pduA-X mutation had been restored by introducing the intact pdu operon through transduction. (A-C) Bars represent geometric means ± s.e.m. of the competitive indices. (E) Histopathological changes were scored in sections of the cecum for the experiment shown in Fig 1D. Each bar represents the combined scoring results for one individual animal. *, P < 0.05; **, P < 0.01; MLN, mesenteric lymph node. (PDF) [file ppat.1006129.s002.pdf]

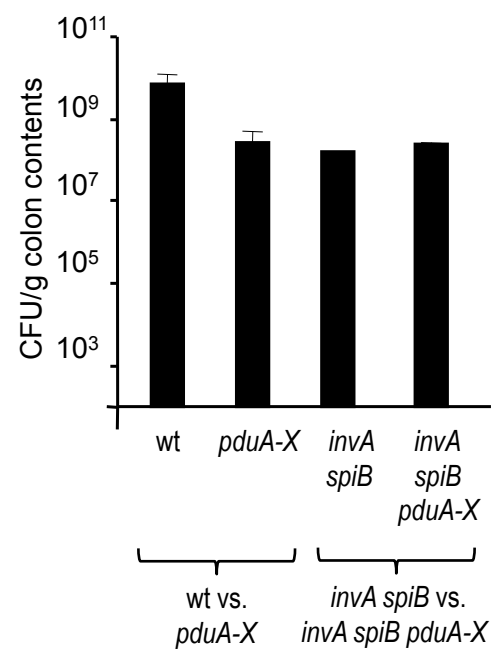

Fig. S3

Supplement: S3 Fig — Streptomycin-treated C57BL/6 mice were infected intragastrically with a 1:1 mixture of the indicated S. Typhimurium strains. Bars represent geometric means ± s.e.m. of the CFU recovered for each strain at 4 days after infection. (PDF) [file ppat.1006129.s003.pdf]
