# Supplementary material for: Respiration of Microbiota-Derived 1,2-propanediol Drives Salmonella Expansion during Colitis
Source: PLoS Pathog. 2017 Jan 5;13(1):e1006129. doi: 10.1371/journal.ppat.1006129 (PMC5215881; doi:10.1371/journal.ppat.1006129)
Supplement: S4 Fig — Criteria for blinded scoring performed by a veterinary pathologist. (PDF) [file ppat.1006129.s004.pdf]

A **Scoring criteria for blinded examination of H&E stained sections from the cecum**

Score	Submucosal edema	Epithelial damage	Exudate	PMN infiltration*	Mononuclear cell infiltration**
0	No changes	No changes	No changes	No changes (0-5)	No changes (0-5)
1	Detectable (<10%)	Desquamation	Slight accumulation	6-20	5-10
2	Mild (10%-20%)	Mild erosion	Mild accumulation	21-60	10-20
3	Moderate (20%-40%)	Marked erosion	Moderate accumulation	60-100	20-40
4	Marked (>40%)	Ulceration	Marked accumulation	>100	>40

* PMN, polymorphonuclear cells

** Number of cells per high power microscopic field

B **Histopathology scoring scheme**

Total score	Description
>8	Severe inflammation
6-8	Moderate inflammation
3-5	Mild inflammation
0-2	normal

Fig. S4
